# Process Evaluation and Experience Sharing on Utilizing Information Communication Technologies and Digital Games in a Large Community Family Health Event: Hong Kong Jockey Club SMART Family-Link Project

**DOI:** 10.3389/fpubh.2020.579773

**Published:** 2020-12-22

**Authors:** Shirley M. M. Sit, Agnes Y. K. Lai, Tai-on Kwok, Hoi-wa Wong, Yiu-lun Wong, Eliza Y. W. Lam, Judy Y. W. Chan, Florence S. W. Kong, Kerin Cham, Charles K. K. Ng, Teresa Yip, Terry S. Y. Tsui, Chiu-man Wong, Bell C. L. Wong, Wai-yan Tang, Pui-wah Yam, Macy Chui, Alice Wan, Yu-kwong Kwok, Tai-hing Lam

**Affiliations:** ^1^School of Public Health, The University of Hong Kong, Hong Kong, China; ^2^School of Nursing, The University of Hong Kong, Hong Kong, China; ^3^Technology-Enriched Learning Initiative, The University of Hong Kong, Hong Kong, China; ^4^Caritas-Hong Kong, Hong Kong, China; ^5^Hong Kong Family Welfare Society, Hong Kong, China; ^6^Hong Kong Christian Service, Hong Kong, China; ^7^International Social Service Hong Kong Branch, Hong Kong, China; ^8^Christian Family Service Centre, Hong Kong, China; ^9^The Hong Kong Catholic Marriage Advisory Council, Hong Kong, China; ^10^Hong Kong Children & Youth Services, Hong Kong, China; ^11^St. James' Settlement, Hong Kong, China; ^12^Tung Wah Group of Hospitals, Hong Kong, China; ^13^Hong Kong Sheng Kung Hui Welfare Council Limited, Hong Kong, China; ^14^The Neighbourhood Advice-Action Council, Hong Kong, China

**Keywords:** family happiness, health promotion, information and communication technologies, social services, community event

## Abstract

**Background:** Information communication technologies (ICT) are increasingly used in health promotion, but integration is challenging and involves complex processes. Large community health promotion events are often held but the experiences and processes have rarely been evaluated and published. No reports have described and systematically evaluated an ICT-supported health promotion event using digital games.

**Objective:** We evaluated the development and implementation of a large community family health promotion event with ICT integration to promote family happiness with collaboration between academia (The University of Hong Kong) and the social (family) service sector, and collected feedback from participants and social service workers.

**Methods:** We (i) conducted a systematic process evaluation, (ii) administered an on-site questionnaire survey on participant satisfaction and feedback, and (iii) collected post-event qualitative feedback from social workers on using new technologies, digital game design and overall experiences.

**Results:** Fourteen digital games were designed and run in booths at the event by 12 non-governmental social service organizations and academia. Four gaming technologies were utilized: chroma key (green screen), somatosensory (kinect and leap motion techniques), augmented reality and virtual reality. 1,365 participants joined the event, in which 1,257 from 454 families were recruited and pre-registered through 12 NGOs. About 39.3% were male and more than half (53.3%) were aged 18 years and above. About 3,487 game booth headcounts were recorded. Games using virtual reality, kinect motion and green screen technologies were most liked. The average game satisfaction score was high (4.5 out of 5). Social service workers reported positive experiences with using new technologies in health promotion, and interests in future collaborations involving more ICT.

**Conclusions:** Our systematic evaluation showed successful integration of ICT components in the health promotion event. This event, most likely the first of its kind, served as a capacity building and knowledge transfer platform for interdisciplinary co-sharing and co-learning of new technologies. It provided a solid foundation for further academic and social service partnerships and should be a useful model for similar community events and their evaluation. Further development and integration of ICT for health promotion among social service organizations with comprehensive evaluation are warranted.

## Introduction

Advances in information communication technologies (ICT), encompassing all digital technologies that facilitate the electronic capture, processing, storage, and exchange of information (1), provide unique opportunities for novel, innovative ways to deliver and promote public health messages and interventions. Despite known advantages and enthusiasm for new technologies in health promotion, embedding ICT into interventions is challenging and involves complex processes and changes at different levels among various stakeholders (2, 3), with close collaboration and full transparency. The successful implementation of community-based health promotion interventions also relies on stable leadership, and effective communication and partnership among and within agencies (4, 5). Adoption of ICT and digital technology in social services is uncommon (6–8). We searched PubMed, Cochrane Library and Social Care Online using keywords including “public education,” “health promotion,” “event,” “ICT,” “digital technology,” “social services,” and “process evaluation,” and found no existing studies or reports, up to November 27, 2020, describing an ICT-supported community health promotion event using digital games with process evaluation.

The Hong Kong Jockey Club SMART Family-Link (JCSFL) Project^1^ is a four-year (2018–2021), inter-disciplinary, cross-sectoral academic and social (family) service collaboration initiated by the Hong Kong Jockey Club Charities Trust with a donation of over HK$157 million (USD$1 = HKD$7.8). The project's primary aim is to advance the use of ICT in family services to enhance service efficiency and effectiveness and promote family well-being in Hong Kong. Project partners include the School of Public Health (SPH) and Technology-Enriched Learning Initiative (TELI) of The University of Hong Kong (HKU), and 26 Integrated Family Service Centers (IFSCs) and Integrated Service Centers (ISCs) operated by 12 non-governmental organizations (NGOs) with funding from the Social Welfare Department (SWD) of the Hong Kong SAR Government. These centers are located throughout all 18 districts of Hong Kong, and provide services to families and individuals from different socioeconomic backgrounds. The use of ICT by these NGO centers was minimal prior to this project.

One of the five main JCSFL project components is public education and advocacy campaigns to increase understanding of healthy family functioning. The project's first public education event was a one-day launch event (LE) on November 11, 2018, utilizing ICT and digital technology to promote family happiness among Hong Kong community participants with academic and social (family) service collaboration. The event was conducted with four objectives: (i) to promote the “Jockey Club SMART Family-Link Project” to the public (9); (ii) to arouse the interests and awareness of social service organizations on using ICT in family services and various JCSFL project components; (iii) to explore the feasibility of integrating digital technology in health promotion implementation and evaluation; and (iv) to promote family happiness, which is an important element of good family functioning and a harmonious society (10, 11). Family happiness is engendered from family activities and closely linked to social capital. Spending time and building connections with family members are pathways to positive family relationships and individual happiness (10). More social interactions with family members and sharing happy experiences can stimulate and enhance family happiness (12). The LE involved close collaboration, for the first time, among SPH, TELI and 12 NGO partners. To foster the collaborative relationship between academia and social services, we co-developed and co-designed 14 digital games utilizing various gaming technologies under the overarching theme of promoting family happiness. Creating and using digital games in non-game contexts can increase interest, motivation and enhance skill acquisition among participants (13–15). Combining gamified tools with a traditional public health initiative can provide a more holistic approach toward improving health (16, 17), and an engaging and enriched experience for participants of all ages.

No reports have systematically described and evaluated the integration of ICT into a large-scale, community family health promotion event. In general, large community health education events are frequently held in Hong Kong but have rarely been vigorously evaluated and we found no evaluation reports published in peer reviewed journals. Such events consume many resources, but the effects are not clear and experiences are not shared. Many of these events are hosted by social service organizations with the primary foci on event implementation and reach. Publications on sharing experiences and evaluation are not the main focus.

The present paper presents our experiences from the academia and social service collaboration in the development and implementation of the event as well as the utilization and integration of ICT tools in the large-scale community family health event using digital games, and the results of a systematic evaluation.

## Methods

### Data Collection Methods

**Figure 1:**
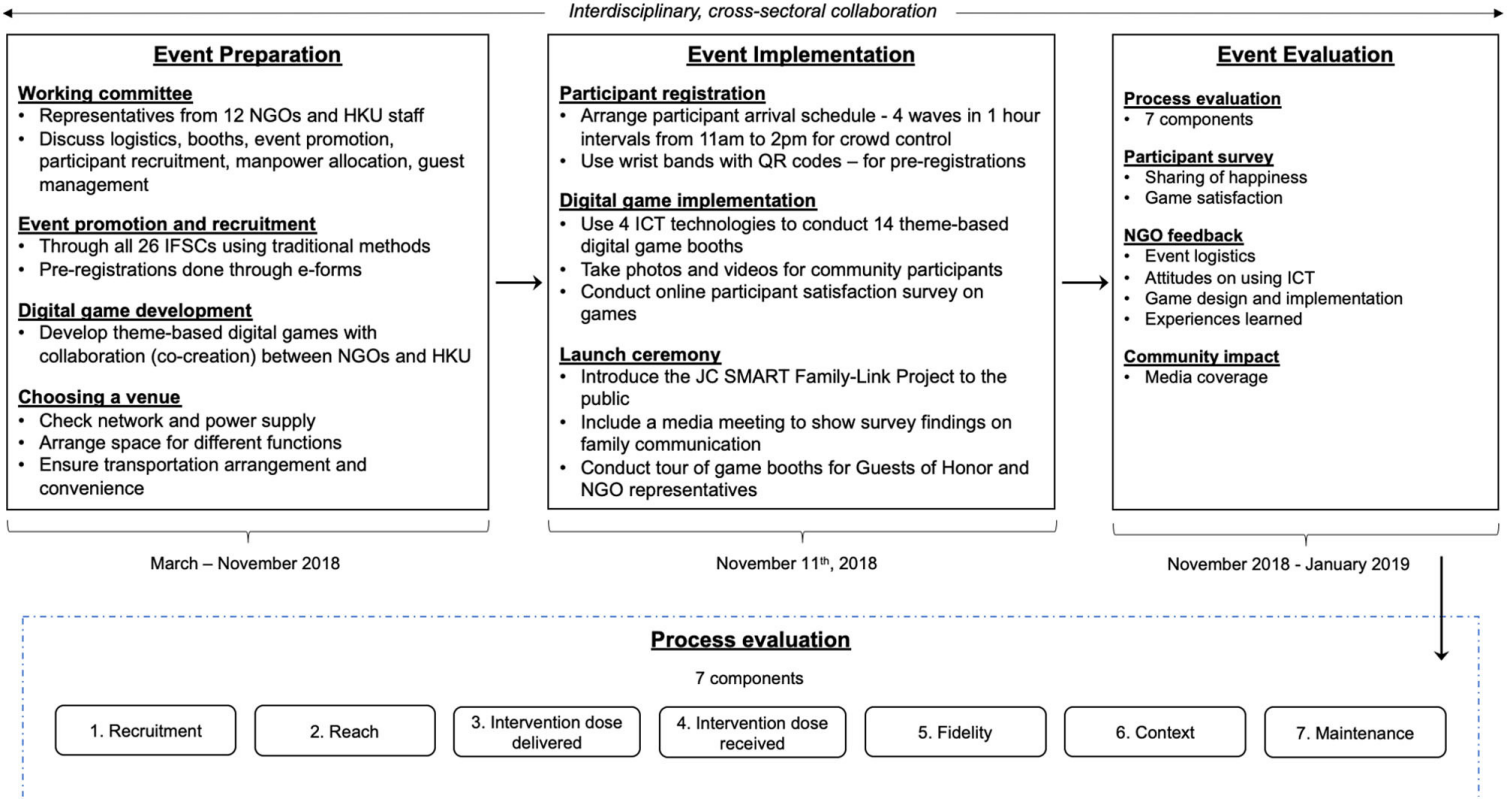
The framework for the preparation, implementation and evaluation of the Launch Event.

We conducted a systematic process evaluation of the event preparation, implementation, and evaluation using seven components: recruitment, reach, intervention dose delivered, intervention dose received, fidelity, context and maintenance (Figure 1). All participants were invited to answer a simple questionnaire survey during queuing and immediately after playing the digital games.

Qualitative on-site feedback was collected *via* 5-min semi-structured interviews immediately after completion of the event from 12 NGO representatives, who were each responsible for a game booth. Three theme-based questions were asked: (i) “What is your experience and overall feedback on this event and your observations of participants?”; (ii) “What experiences did you have with the digital games and new gaming technologies?”; and (iii) “What have you learned about using ICT and how confident are you on integrating ICT in the future?” Feedback was compiled and collated in an Excel spreadsheet, and organized by the three themes. On-site, unobtrusive observations were made by HKU staff and recorded to corroborate the findings from the interviews. On-site written and verbal consent were obtained from both community participants and social workers, respectively.

### Event Preparation

#### Working Committee

A working committee was first formed in early 2018 with HKU staff and representatives from each participating NGO. Details concerning LE objectives and logistics, booth elements, event promotion and recruitment, manpower allocation and guest invitation and management were discussed in subsequent meetings throughout May to October 2018 and unanimously agreed upon. These processes were crucial for planning and framing the event logistics.

#### Event Promotion and Recruitment

Event promotion and recruitment was conducted through all 26 IFSCs. Each IFSC was given a participant quota of 50 participants. This quota was decided and agreed upon by all NGOs according to the time and capacity of the venue to avoid overcrowding and long queues, and the seating capacity of buses to bring participants to the venue. NGOs proceeded with familiar traditional promotional methods, including placing posters in centers and by word-of-mouth. HKU prepared simple online e-registration forms for each IFSC. Each family was registered as a unit with a maximum of four members. The member registering on behalf of the others (most often a parent) was identified as the principal participant for that family. The relationships of family members with the principal participant were recorded in the registration form. HKU prepared wristbands for all pre-registered participants from each respective center prior to the event day. Wristbands for each family were labeled with a QR code, a unique family ID and the principal participant's surname for easy identification.

#### Digital Game Development

HKU team members formed individual working groups with each NGO partner for the co-creation, co-development and co-design of customized, theme-based digital games to integrate concepts of positive family well-being to enhance family happiness (Table 1; names of NGOs are not disclosed and will be represented by letters throughout this paper. Anonymity was ensured to avoid comparison among NGOs). Game themes were directly referenced from the five JCSFL themes, including SMART Parenting, SMART Emotion, SMART Coping, SMART Communication and SMART Living Habits. HKU introduced and demonstrated four simple and readily available technologies, and also showcased two games designed and developed by HKU for the LE. Four gaming technologies were presented, including chroma key (green screen), somatosensory (kinect and leap motion techniques), augmented reality (AR) and virtual reality (VR). Each NGO provided insight and specific requests for the development of a digital game catered to their unique service needs and overall service user profiles. The duration of the games were designed to be 1–2 min to reduce queue time. Gameplay feasibility, revisions and feedback were discussed in subsequent meetings, with each digital game finalized at the end of October 2018.

**Table 1:** Summary of digital games used in the launch event.

Booth^*^	**Gaming technology^#^**				**Average gameplay duration (min)**	**Total number of headcounts**	**Satisfaction score^∧^**	**% of participants that rated “4” and “5”**
	**Chroma key**	**Somato-sensory**	**AR**	**VR**				
A	✓				1	151	4.46	87.11%
B		✓			2	371	4.46	87.98%
C	✓			✓	2	270	4.78 (2nd)	95.91%
D			✓		3	196	4.46	88.00%
E				✓	3	196	4.67	94.62%
F^+^		✓			3	194	4.75 (3rd)	97.33%
G^+^	✓				2	325	4.18	80.14%
H	✓				1	254	4.56	90.35%
I	✓				1	259	4.55	92.66%
J		✓			1	262	4.28	82.50%
K		✓			1	300	4.97 (1st)	99.62%
L		✓			2	145	4.09	73.42%
M			✓		2	252	4.04	75.15%
N		✓			2	312	4.60	91.61%
Average					2	249	4.49	88.3%

* *Game booth descriptions*.

*A: Promoting family happiness by taking fun family photos using props and backgrounds*.

*B: Promoting physical activity and family happiness by playing an “escape from the dinosaur” challenge*.

*C: Promoting family happiness by taking fun family photos with a virtual background*.

*D: Promoting family communication by drawing and writing messages of appreciation to family members*.

*E: Promoting positive family communication by gathering happy sayings for family member*.

*F: Promoting family communication and happiness by writing or drawing out expressive messages*.

*G: Promoting family communication by taking fun family photos and posing next to a virtual dinosaur*.

*H: Promoting family communication by taking fun family photos with different backgrounds*.

*I: Promoting family communication by taking fun family photos framed with different family-related blessings*.

*J: Promoting physical activity by competing at timed fitness challenges*.

*K: Promoting physical activity and family happiness by playing a basketball shooting challenge*.

*L: Promoting positive family communication by playing a cooking challenge*.

*M: Promoting family communication by building a virtual dream home*.

*N: Promoting family communication and happiness by recording expressive audio messages*.

# *4 gaming technologies*.

**1. Chroma key:** using visual effects (green screen) technique to layer two images or video streams together based on color hues.

**2. Somatosensory:** using Kinect and Leap motion techniques to obtain a very informative description of the hand, body, and foot pose that can be utilized for accurate gesture recognition.

**3. Augmented reality:** using technological devices to enrich the real environment with virtual objects, running in real time.

**4. Virtual reality:** allowing participants to have a complete immersive experience that shuts out the physical world and interact in real-time in a 3-dimensional environment.

+ *First two games developed for the LE by HKU*.

∧ *Satisfaction score ranged from 1 = not so satisfied to 5 = very satisfied*.

#### Choosing a Venue

Three primary factors were considered in choosing an appropriate venue for the event: an indoor location (i) with a stable network and power supply, (ii) with sufficient space for at least 14 game booths and different functions such as guest reception, rest areas, child care rooms, and mobile phone charging stations, and (iii) that is conveniently located with a family-friendly atmosphere. Network availability and stability was crucial, as the LE involved several ICT components that required a speedy and stable internet connection. A final decision was made in August 2018 to host the LE on the lower ground floor of the Centennial Campus at HKU (Supplementary Figure 1). We also had a contingency plan in case of network and connectivity issues. We conducted site visits for planning prior to the event during August to October 2018.

### Event Implementation

Event setup was conducted 1 day before the LE. HKU team members were given a full briefing on the event rundown and logistics. The event had two parts—digital game booths and a launch ceremony.

#### Participant Registration

There were two types of participant registration: (i) the main type was for pre-registered community participants from IFSCs, and (ii) some walk-in individuals on the event day, as the campus was open to all and the event would attract some visitors and passers-by. HKU prearranged transportation for participants of each IFSC to arrive to the venue. In order to exercise crowd control and ensure participants had a comfortable and enjoyable experience, an arrival schedule from 11 am to 2 pm with four arrival waves in 1-h intervals was devised. Buses from different NGOs would drop off and pick up participants on a predetermined schedule. Upon arrival, NGO representatives liaised with HKU team members and collected envelopes containing the wristbands that HKU had prepared in advance. Unique QR codes were located on each wristband for efficient registration and souvenir redemption. A registration booth was located at the entry of the venue for interested individuals to register as walk-in participants, who received wristbands generated on the spot.

#### Digital Game Implementation

The digital game booths were arranged in the venue so that similarly themed games were placed together. Twelve booths were hosted by individual NGOs while the remaining two were hosted by HKU. Supplementary equipment, including laptops, television screens, iPads, and digital game props, were prepared in advance and provided to NGOs by HKU. Participant wristband QR codes were scanned prior to joining each game. Instructions were given by staff members and photos and short videos were taken during gameplay and sent back to participants via a unique link paired with their QR codes. An online questionnaire was also linked to the QR codes to collect their feedback on the games and to track game booth headcounts and other data.

#### Launch Ceremony

A launch ceremony was held at 2 pm to introduce the JCSFL project to the public and present the findings from a territory-wide random sampling telephone survey with more than 1,600 local residents on ICT use in family communication in Hong Kong. Survey respondents who used family e-chat groups and those who used these groups more frequently had higher subjective happiness scores, family communication scores and family health, happiness and harmony scores (18). These results supported the JCSFL's aims to make good use of ICT to promote family communication and health, happiness and harmony, and strengthen family relationships.

VIP guests, among others, included the Hong Kong SAR Government's Secretary for Labor and Welfare and senior officers of SWD, who have been supporting the JCSFL project since its inception and were particularly interested to learn about using ICT games in family services. Guests from the social service sector and the media were also invited. The ceremony also served as a break time for NGO workers and HKU staff operating the game booths. The VIP guests had a guided tour of the booths and tried some games after the ceremony.

### Event Evaluation

#### Process Evaluation

A systematic evaluation was conducted according to seven components of process evaluation (19, 20), including *recruitment* (procedures used to approach and attract participants), *reach* (measure of participation and characteristics of participants), *intervention dose delivered* (amount or number of intervention units delivered by interventionists), *intervention dose received* (extent in which participants actively engaged and interacted with interventionists and other participants, as well as their satisfaction toward the intervention and interactions with staff), *fidelity* (extent to which intervention was implemented as planned), *context* (environmental issues that might affect intervention implementation or study outcomes) and *maintenance* (long-term sustainability of intervention and outcomes). These seven components helped guide the conceptualization, development, implementation and eventual evaluation of the program.

#### Participant Survey

Since participants were accustomed to completing questionnaire surveys after center-based programs, but not used to answering surveys while attending community events usually conducted in public areas, the preloaded questionnaire on iPads was very short. Before playing the game (T1) while queueing, participants (both adults, and children with parental consent) were first asked one question on whether they had shared happy things with family in the past week (Yes or no). Immediately after completing each game (T2), participants were asked to rate their satisfaction by the question, “How satisfied are you with this game?” Responses were made on a five-star scale (1 = not satisfied to 5 = very satisfied). On-site, unobtrusive observations of participants (including VIP guests who tried the games) were also made by HKU staff stationed at different game booths around the venue.

#### NGO Feedback

HKU staff visited each game booth to obtain post-event feedback from NGO workers on event logistics, game booth implementation, their attitudes toward incorporating digital games and ICT elements into their services, and overall experiences learned. NGO workers were encouraged to visit other game booths to share experiences.

#### Community Impact

A media coverage summary was collated after the LE was completed and later disseminated to the public via the JCSFL website.

## Results

### Process Evaluation

#### Recruitment

Prior to the LE, all IFSCs preferred registration using paper and pen; online registration was never used before. With some encouragement, all 12 NGOs took the first step in ICT integration by using online e-registration forms prepared by HKU. HKU helped complete the e-registration process by compiling the final list of participants and preparing wristbands, which were disseminated to NGO representatives on the event day when they arrived with participants via buses. This LE thus provided NGOs the first experience of the ease and utility of online registration methods.

#### Reach

The event reached a total of 1,365 participants, in which 1,257 from 454 families were recruited and pre-registered through 26 IFSCs of 12 NGOs. Seventeen out of 26 IFSCs exceeded the 50 participant quota. On the day, 108 individuals registered as walk-in participants at the venue. Due to limited manpower and as walk-in participants were not the main targets, their demographic information was not collected. Table 2 shows that 39.3% were male and more than half (53.3%) were aged 18 years and above. Among these adults, 28.5% were male. Majority of family members were either the principal participant's child or grandchild (46.7%) or their spouse (31.5%).

**Table 2:** Characteristics of 1,257 IFSC-registered participants.

***n* = 1,257**	***n* (%)**
**Sex**	
Male Female	494 (39.3) 763 (60.7)
**Age groups, years**	
<18 18–29 30–49 50–64 ≥65	587 (46.7) 47 (3.7) 491 (39.1) 84 (6.7) 48 (3.8)
Relationship of family members with principal registered participant^*^	
Spouse Children/Grandchildren Parents/Grandparents Siblings Cousin or other relatives Not mentioned	396 (31.5) 587 (46.7) 97 (7.7) 36 (2.9) 75 (5.9) 66 (5.3)

* *Participating family member that registered for the event on behalf of other members*.

#### Intervention Dose Delivered and Received

**Figure 2:**
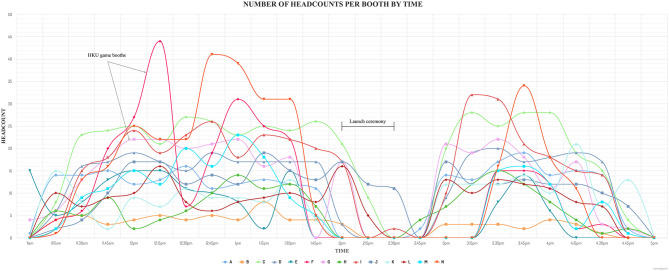
Number of game booth headcounts by time (*n* = 3,487).

**Figure 3:**
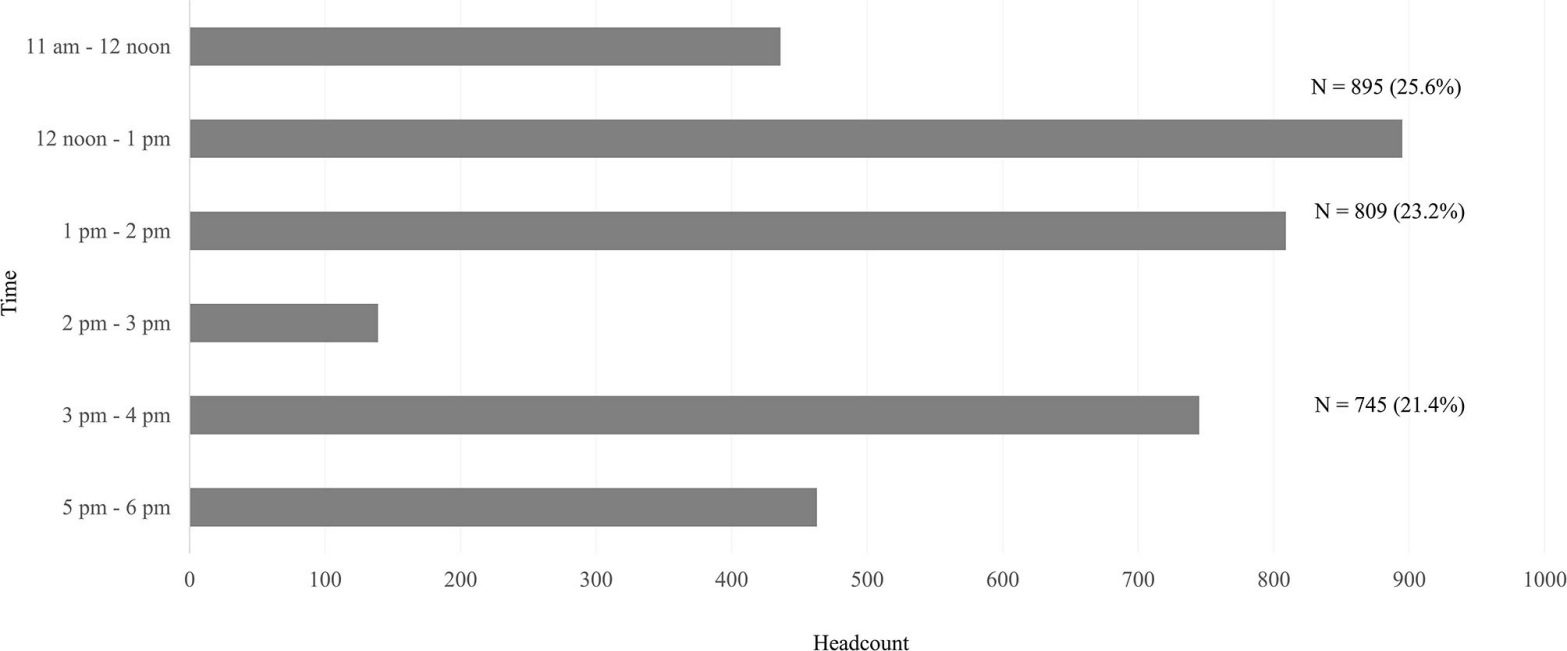
Headcounts of participants joining games by time frame (*n* = 3,487).

The average gameplay duration for the game booth ranged from 1 to 3 min (Table 1). This did not include queue time or game briefing. Queue time was short (about 2 to 10 min) as having 14 game booths allowed for shorter queues. The wristband QR codes recorded 3,487 headcounts for all booths. The highest number of headcounts was for booth B (*n* = 371), followed by booth G (*n* = 325), and booth N (*n* = 312). QR codes recorded headcounts for each booth by time (Figure 2). The number of headcounts at around 2 pm to 2:30 pm decreased due to the launch ceremony (Figure 3). The spikes in headcounts for Booth F and Booth N were due to the location of the booths being near the arrival area. The peak time was 12 noon to 1 pm (25.6%), followed by 1 pm to 2 pm (23.2%), and 3 pm to 4 pm (21.4%). The average number of participants per game was 249 ± 66 (mean ± SD) (range: 145 to 371). The average number of games each participant played was 3.8 ± 2.4 (range: 1 to 18).

Three thousand seven hundred ninety two photos and 185 videos were taken for participants by HKU staff during gameplay using iPads, and 524 photos were taken by HKU staff through the game system. All photo images and videos were sent to the participants and their corresponding IFSCs both through their individual QR codes and email. Souvenirs, including mobile phone stands and charger cases, were available for redemption before leaving the venue via QR code scanning.

#### Fidelity

All 14 games were implemented successfully with no major issues. The network was stable and allowed for different game components and technologies to operate as planned. The wristbands and QR codes worked effectively and successfully captured and recorded participants' feedback *via* the online questionnaires. Some participants were not able to play all the games because they had to catch buses pre-arranged by their respective IFSCs. A few games had longer queues so additional manpower was needed. One NGO gave out cards with different time slots to facilitate better crowd management. Since VR glasses are not recommended for children under 13, younger participants were unable to join VR-supported booths and this resulted in some disappointment.

#### Context

Hosting a large-scale event in an academic setting had several advantages. The venue was equipped with a stable network and power supply and had sufficient space for all event-related functions, including the launch ceremony with a spacious stage and more than 100 seats. It was conveniently located with many transportation options to and from the venue, and the indoor location with air conditioning was comfortable. The venue was also in close proximity to HKU staff offices, which helped facilitate event implementation including ease of setup and having additional resources nearby such as technical assistance and staff support. Holding the LE at the oldest tertiary institution in Hong Kong was also an attractive factor for participants. The Centennial Campus—a new campus at the university completed in 2012 and attached to a mass transit railway station—was an attractive and freely accessible site for participants to visit from all over the city, and made a nice outing for parents and children. The 108 walk-in participants were more than expected.

#### Maintenance

**Figure 4:**
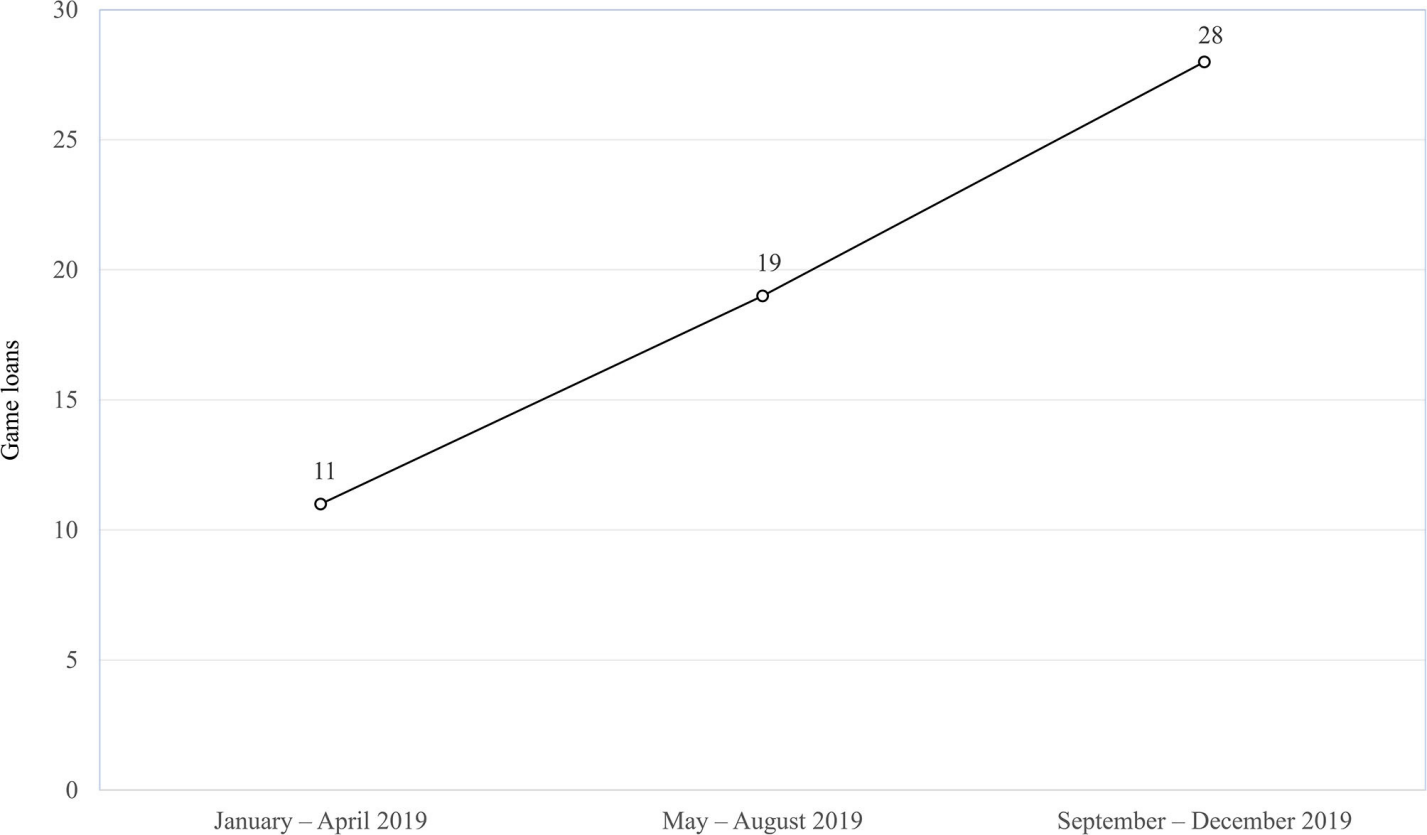
Number of game loans by NGOs by time (*n* = 58).

Photos and videos taken of participants with their consent during the event were linked to unique QR codes printed on cards and disseminated to them before they left the venue. Participants interested in learning more about the JCSFL project and promoting family happiness were given the link to the official project website that was soon to be launched after the completion of the event. To foster sustained collaboration with NGOs, HKU developed an online game equipment loan system soon after the LE in December 2018. IFSCs could submit requests to borrow equipment and supplies for the developed games for an agreed upon period depending on equipment availability. Learning materials regarding game details and logistics were also uploaded to the JCSFL project's worker capacity building and knowledge transfer e-platform, a project component accessible to all social workers of partnering NGOs. As a result, all 14 games continue to be adapted and used by different NGOs in their respective centers and implemented into their own activities and programs. The first game loan request was made in January 2019, and a total of 58 requests were made from different IFSCs by the end of December 2019. Figure 4 shows the number of loans gradually increased with time, indicating NGOs became more familiar and comfortable with integrating ICT into their own programs. HKU has also utilized these games in other JCSFL public education events to promote family well-being. Increased use of games by NGOs and HKU has allowed for continuous feedback and subsequent game revisions and improvements made by HKU.

### Participant Survey

Of 1,302 survey responses, 88% of participants reported they had shared happy things with family in the past week. The average game booth satisfaction score was 4.5 (out of a five star rating). Table 1 shows the highest satisfaction score was for booth K (4.9), followed by booth C (4.8), and booth F (4.7). Supplementary Figure 2 shows the descriptions, equipment lists and pictures of the digital games used in different game booths. The results showed that somatosensory games using Kinect motion techniques, games using VR technology and chroma key green screen techniques, as well as family-based activities were the most liked. The unobtrusive observations made by HKU staff showed high enthusiasm, engagement and happiness from participants of all ages. Our VIP guests also showed great interest in and appreciation for the games.

### NGO Feedback

NGO social workers reported overall positive experiences with the LE, specifically on three aspects including (i) overall event and participant feedback, (ii) digital game design and experience with gaming technologies, and (iii) ICT utilization and integration.

Workers expressed a great appreciation for the opportunity to test out new technologies with the support of HKU staff and they learned a lot from other social workers. Many also reported that using ICT, such as QR codes and online surveys, was easier than expected. They thought the photo-taking component was a very worthwhile addition to the booths, and observed that many families enjoyed it very much. Some of their words are quoted below.

“*The launch event was organized well and a rare opportunity for my colleagues and I to test out different ICT components that we've never tried before. This learning experience was something we had previously wished for… it finally became a reality through this event.”* (NGO social worker, female)

“*Our service users, especially families with elderly participants, really enjoyed taking photos at different game booths, and was happy that they could receive them electronically for further sharing with family members and friends… this was a special opportunity for them.”* (NGO social worker, female)

Many were impressed with the VR technology, but reported that such games had longer queues and turnaround time due to high levels of interest and relatively longer preparation time (such as limited number of VR headsets and time spent to adjust the headsets). They observed that many participants, especially children, were captivated over the fully immersive experience of a 3-dimensional world with different locations, and that many participating families were of lower socioeconomic status and had rarely traveled abroad. These booths therefore required additional manpower and effort to ensure smooth logistics. Workers responsible for hosting VR-supported booths noted disappointment in younger participants who were advised against joining due to the age restriction on using VR glasses. A few workers also reported that their Kinect systems were not sensitive enough to participant movements and had to be restarted sometimes. This was most likely due to too many people around the booths at times, which made it difficult for the systems to differentiate between different people and movements nearby.

“*We were able to test out different aspects of our game and identify areas… that needed improvement… because our game was not as interactive and high-energy as other booths, it was less attractive to participants, especially children. There is room for improvement in terms of game design… I appreciate that the game can be further revised and adapted to our specific needs through feedback and collaboration.”* (NGO social worker, male)

Many workers suggested to use larger QR codes on wristbands in the future to improve and shorten turnaround time and enhance workflow. They observed that some participants still preferred the traditional method of stamp collection for souvenir redemption over QR codes as they had no prior experience using such technology. Some workers also suggested that additional training and support for staff was needed to facilitate further integration of different ICT components in their own programs and services.

“*This is the first time our workers have had first-hand experience using VR technology… more resources and training to help us familiarize with such technologies would be appreciated.”* (NGO social worker, female)

Due to the overall positive experiences, many workers suggested HKU host another similar collaborative event in the future for further knowledge transfer and capacity building.

### Community Impact

Media coverage of the event included 44 online and print newspapers/publications and television broadcasting clips in the 2 weeks after event completion. The coverage summary was published on the JCSFL website with publication names, dates, and relevant links for online articles and video clips^2^.

## Discussion

Our one-day LE was successfully launched as the JCSFL project's first public education event using digital games on a university campus with deep academic (public health and teaching technology) and social service (social work and family service) collaboration to promote family happiness in participants from the community. This event, probably the first in the world, served as a capacity building and knowledge transfer platform for interdisciplinary co-sharing and co-learning of new technologies and skills between academia and social services. It also facilitated communication and sharing among different NGOs, which had very limited contact with each other prior to this event. The success of testing and demonstrating ICT application in game development and program implementation such as QR codes to collect instant participant feedback raised the interests of social workers on ICT use in family service program design, planning, participant recruitment and evaluation. The use of QR codes and gaming technologies are new and innovative strategies that can be integrated into family services in Hong Kong. The design and planning for subsequent JCSFL public education events and programs, as well as collaborative NGO programs have been based on experiences learned from the LE. Hence, the present paper should be a useful model or reference for similar community health events elsewhere.

The event was also a showing of solidarity—it was the first time all 12 NGOs were under one roof participating and sharing in the same event to promote the JCSFL and their centers. Hosting the event on the university campus also showcased HKU's leading role in uniting 12 non-governmental social service organizations and the community. Through this event, we are the first to demonstrate a successful community-academic partnership of a health promotion event utilizing digital technology. This event has laid a solid foundation for further collaboration among community-based social service organizations, and between them and academics in the context of the JCSFL project and beyond.

The LE provided frontline workers from the social service sector hands-on practical experience on how ICT can help enhance and streamline their work and service planning for increased efficiency, community reach and worker capacity. Experiences and challenges from the event have helped facilitate the rapid increase in integration of ICT-supported digital games in different centers, programs and activities with service users of all ages, and have helped build confidence among social workers. By empowering them to implement these new skills and technologies learned into their own future programs, the positive effects of the community-academic partnership can extend beyond just this single event. The potential of ICT for improving the self-efficacy of health promotion initiatives comprises three factors: personalisation, adaptation and mobility (21). A major breakthrough can thus be seen through the subsequent game loaning records that show NGOs have been improvising and adapting ICT components from the LE to fit their specific service needs and programs.

To the community participants, the LE provided parents and children fun experiences from playing various games together with new gaming technologies they had not experienced before. We emphasized and assisted them to share memorable experiences by using ICT to share photos and videos with other family members who did not attend the event so that they could foster more family happiness. As family happiness can be generated from family activities and spending time together with family members (10, 12), the success of the event and the high satisfaction scores were indications that digital games and integration of ICT in services could help families build connections, increase social interactions and enhance happiness. Through this event, we were able to establish a vast community network and could attract participants and NGO workers later to follow our project Facebook page and subscribe to the project's Family Portal^3^ for information and tools, and participation in future events when these sites are launched. The percentage of male adult participants in the LE was 28.5%, compared to <20% in past health promotion events and interventions conducted by our team members (22–24). This relative increase in the proportion of men, who are more difficult to reach, by about 50% (absolute increase by 10 percentage points) could be due to the appeal of ICT and digital games in our event.

This event required strenuous effort and many resources from all collaborators. Strengths of this study included the diversity and number of participating stakeholders, staff and volunteers, as well as the close partnerships between academia and community. We had a highly skilled team of game developers and engineers from TELI who were able to help NGOs co-create complex games. The event had 14 games so queuing was kept to a minimum. We were also able to receive simple, instant feedback from our participants and NGO partners, and the questionnaire data could be analyzed without coding and data input.

The technological shift of increased use of ICT in health services and promotion is helping to reach and attract a wider audience, while also increasing the capacity for health interventions to be personalized and adapted to the distinctive and dynamic needs of individuals and communities (21, 25, 26). Thus, ICT can enhance the delivery, implementation and success of traditional public health initiatives and improve the efficacy of health promotion (21, 27). Furthermore, the World Health Organization has consistently called for the development and mainstreaming of new technologies and innovations in health services to address population health and meet local and global challenges (28). The present paper can make a valuable contribution to the literature on ICT-supported program and large community event development and evaluation. Our systematic evaluation showed that the event was delivered as planned with satisfactory results.

Our study had several limitations. Firstly, most of the participants were parents with young children. The results might not apply to older people. Secondly, the duration for each game was also short and the effect of each one would be limited and could not be measured individually. Thirdly, the location of the LE was inconvenient for some participants. Because travel time was too long, the time on games was too short. Finally, we did not assess the outcome of family happiness and longer-term effects on participants. In future community events, we plan to conduct both quantitative and qualitative outcome evaluation on family happiness and related outcomes with follow-up assessments.

## Conclusions

The present systematic evaluation of a large-scale community family health promotion event with deep collaboration between academic and social (family) service organizations utilizing ICT to promote family well-being in Hong Kong showed successful integration of ICT components in game development, launching of the event and program evaluation with encouraging results. The quantitative and qualitative findings demonstrated high satisfaction, active engagement and positive feedback from both community participants and NGO social workers. The success of the event has provided a solid foundation for further community and academic partnerships and utilization of ICT in social services, and a useful model or reference of similar events and their evaluation in China and elsewhere. Further development and deeper integration of ICT for health promotion among social service organizations with comprehensive and vigorous evaluation are warranted.

## Trial Registration

The research protocol was registered at the National Institutes of Health (identifier number: NCT03993951) on June 2nd, 2019.

## Data Availability Statement

The datasets presented in this article are not readily available because the sharing of data to third parties was not mentioned in the consent form for participants. Requests to access the datasets should be directed to Agnes Y. K. Lai, agneslai@hku.hk.

## Ethics Statement

This study involving human participants was reviewed and approved by Institutional Review Board of the University of Hong Kong/Hospital Authority Hong Kong West Cluster. Written informed consent to participate in this study was provided by the participants' legal guardian/next of kin.

## Author Contributions

AL, MC, AW, Y-kK, and T-hL contributed to the conception and design of the study. AL, T-oK, H-wW, Y-lW, EL, JC, FK, KC, CN, TY, TT, C-mW, BW, W-yT, and P-wY contributed to the implementation of the program. H-wW organized the database. SS and AL analyzed the data. SS wrote the first draft of the manuscript. SS, AL, and T-hL critically reviewed and revised the manuscript. All authors read and approved the final manuscript.

## Conflict of Interest

The authors declare that the research was conducted in the absence of any commercial or financial relationships that could be construed as a potential conflict of interest.

## Footnotes

^1^https://www.jcsmartfamilylink.hk/

^2^https://www.jcsmartfamilylink.hk/blog/clipping-report/

^3^www.jcsmartfamilylink.hk/family-portal

## Abbreviations

ICT: information communication technologies
JCSFL: Hong Kong jockey club SMART family-link
SPH: school of public health
TELI: technology-enriched learning initiative
HKU: The University of Hong Kong
IFSCs: integrated family service centers
ISCs: integrated service centres
NGOs: non-governmental organizations
SWD: social welfare department
LE: launch event
AR: augmented reality
VR: virtual reality.
