# Supplementary figures and images for: Process Evaluation and Experience Sharing on Utilizing Information Communication Technologies and Digital Games in a Large Community Family Health Event: Hong Kong Jockey Club SMART Family-Link Project
Source: Front Public Health. 2020 Dec 22;8:579773. doi: 10.3389/fpubh.2020.579773 (PMC7783326; doi:10.3389/fpubh.2020.579773)

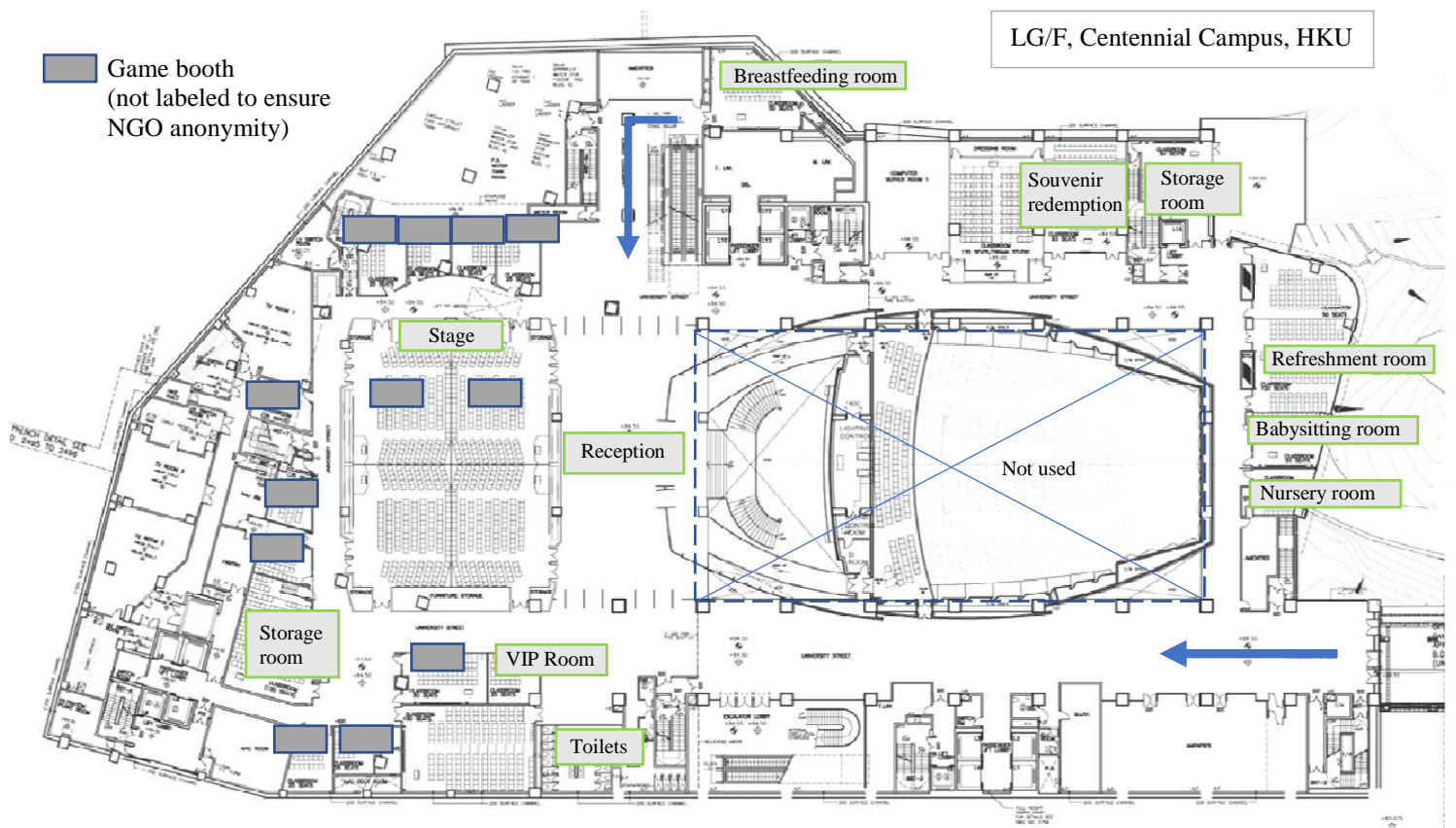

**Supplementary Figure 1.** Floor plan of the Launch Event

Supplement: Supplementary file 1 [file Data_Sheet_1.PDF]
