# Supplementary material for: Process Evaluation and Experience Sharing on Utilizing Information Communication Technologies and Digital Games in a Large Community Family Health Event: Hong Kong Jockey Club SMART Family-Link Project
Source: Front Public Health. 2020 Dec 22;8:579773. doi: 10.3389/fpubh.2020.579773 (PMC7783326; doi:10.3389/fpubh.2020.579773)
Supplement: Supplementary file 2 [file Data_Sheet_2.PDF]

Supplementary Figure 2. Descriptions, equipment lists and pictures of digital games used in different game booths

Game booth	Description
**A**	Promoting family happiness by taking fun family photos using props and backgrounds
**B**	Promoting physical activity and family happiness by playing an 'escape from the dinosaur' challenge
**C**	Promoting family happiness by taking fun family photos with a virtual background
**D**	Promoting family communication by drawing and writing messages of appreciation to family members
**E**	Promoting positive family communication by gathering happy sayings for family member
**F**	Promoting family communication and happiness by writing or drawing out expressive messages
**G**	Promoting family communication by taking fun family photos and posing next to a virtual dinosaur
**H**	Promoting family communication by taking fun family photos with different backgrounds
**I**	Promoting family communication by taking fun family photos framed with different family-related blessings
**J**	Promoting physical activity by competing at timed fitness challenges
**K**	Promoting physical activity and family happiness by playing a basketball shooting challenge
**L**	Promoting positive family communication by playing a cooking challenge
**M**	Promoting family communication by building a virtual dream home
**N**	Promoting family communication and happiness by recording expressive audio messages

Game A

Promoting family happiness by taking fun family photos using props and backgrounds.

Equipment list:

- Screen display
- Computer [with game program pre-loaded]
- Shooting supplies [includes 4K camera, tripod and green screen]

User experience:

Participants stand in front of a green screen and choose a background with a unique location (such as Rome, Italy) or season (such as a snowy winter) to take a family photo. Photos are sent back to them and their respective IFSCs via email.

玩法

一家人站在綠幕前利用不同的道具和背景，通過電腦合成的方法拍攝一張別出心裁的家庭照。

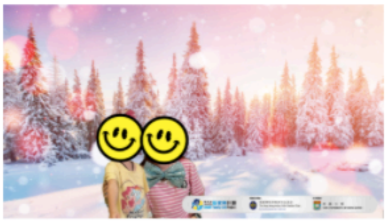

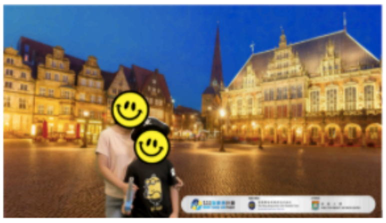

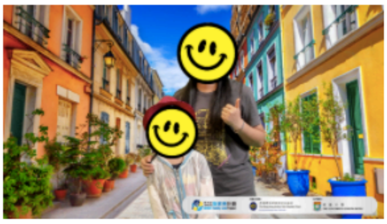

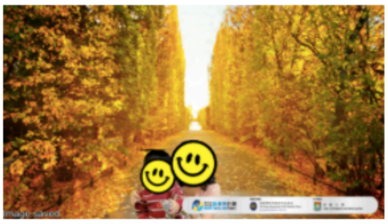

器材	用途
顯示器 [按需要]	顯示器不影響遊戲運作，機構可按實質需要自行配置
電腦 [遊戲程式]	建議在機構的電腦上運行遊戲，如有需要，可與智家樂團隊討論
拍攝用品組裝	包括4K相機、腳架及綠幕組合，用作擷取影象

Game B

Promoting physical activity and family happiness by playing an 'escape from the dinosaur' challenge.

Equipment list:

- Screen display
- Computer [with game program pre-loaded]
- Kinect [sensor that captures body movements]

User experience:

One to two participants stand in front of the Kinect sensor and run in place for a duration of 60 seconds. A progress bar at the bottom of the screen shows the distance needed to successfully escape the dinosaur in the game. The faster the participants run in place, the further they get from the dinosaur. Participants can choose different costumes while queuing to wear during the game, such as a princess or queen, to make the game even more fun. Pictures are taken during gameplay upon their request to capture their happy moments and sent back to them via email.

Remarks:

Game not suitable for frail participants.

玩法

一至兩位家庭成員面向遊戲屏幕，在感應器前快速原地跑步，合作逃離危險的恐龍巢穴。

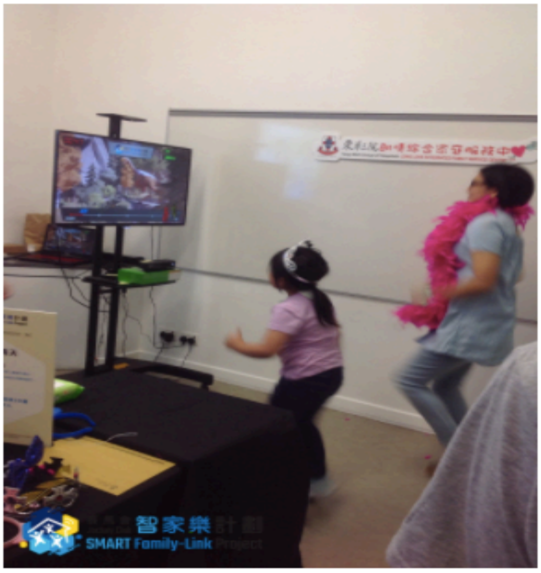

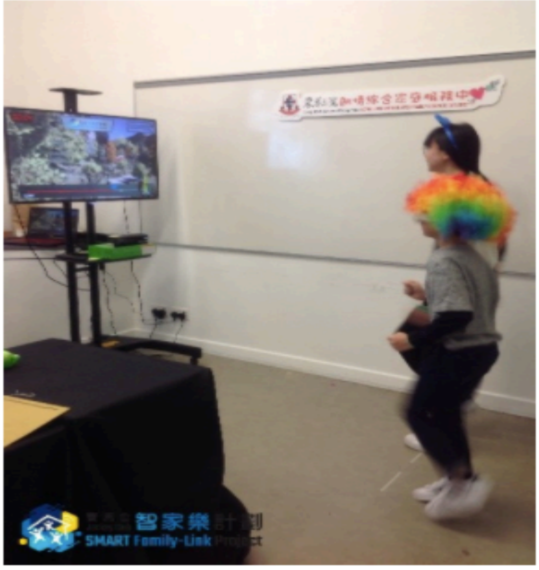

器材

顯示器 [按需要]
電腦 [遊戲程式]
Kinect

用途

顯示器不影響遊戲運作，機構可按實質需要自行配置
建議在機構的電腦上運行遊戲，如有需要，可與智家樂團隊討論
捕捉肢體動作的感應器

Game C

Promoting family happiness by taking fun family photos with a virtual background.

Equipment list:

- Screen display
- Computer [with game program pre-loaded]
- Shooting supplies [includes 4K camera, tripod and green screen]
- Virtual reality device
- Type C extension cable

User experience:

One participant from each family will put on the VR headset, in which they will see different iconic locations from around the world. They will choose one of these locations to take a photo with their family members. The participant wearing the VR headset will experience being in that location, while their family members can see the chosen location on the screen display.

Remarks:

VR headsets not recommended for children under 13 years old. For sanitary purposes, disposable VR masks can be purchased and used.

玩法

一位家庭成員戴上虛擬實境眼罩，利用手上的無線控制器在地圖上選取心儀的地標，然後一家人站在綠幕前通過電腦合成的方法在該地標拍攝一張別出心裁的家庭照。

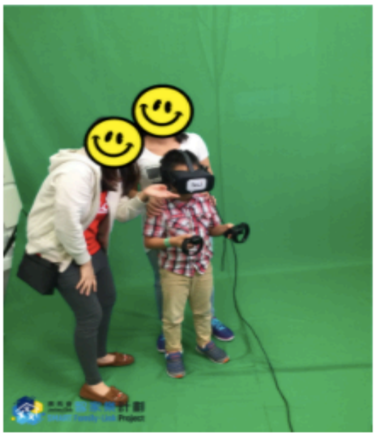

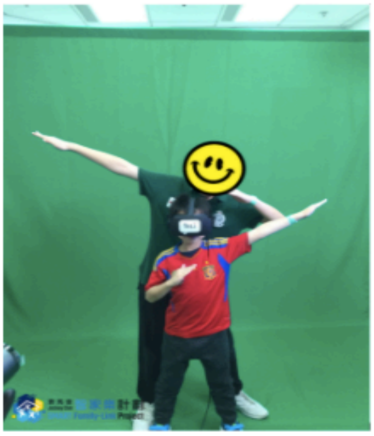

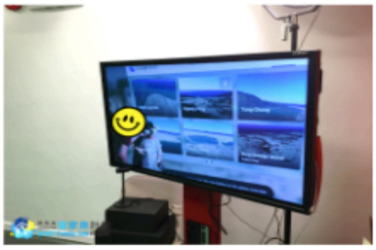

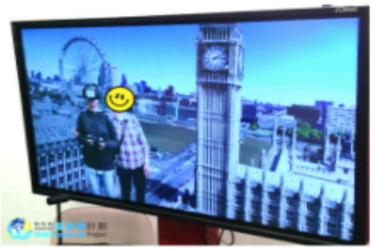

器材	用途
顯示器 [按需要]	顯示器不影響遊戲運作，機構可按實質需要自行配置
電腦 (高階)	電腦上預載遊戲系統，可直接運行遊戲
拍攝用品組裝	包括4K相機、腳架及綠幕組合，用作擷取影象
虛擬實境裝置	遊戲的控制
Type-C擴充器	轉換至不同制式的輸出

<u>Game D</u>

Promoting family communication by drawing and writing messages of appreciation to family members

Equipment list:

- Screen display
- Android tablet [with game program pre-loaded]
- Type C extension cable

User experience:

Participants take turns drawing and writing different messages that express love, appreciation and gratitude towards family members. A snapshot of these messages will be captured as an image and digitally placed on a dandelion, which will then blow into the wind, signifying the carrying of their love and appreciation to their loved ones.

玩法

一家人在輕觸式屏幕上畫出或寫出想對家人表達的心意，完成後寓意忠誠和幸福的蒲公英會帶著這份心意隨風飄送，將祝福帶到遠方。

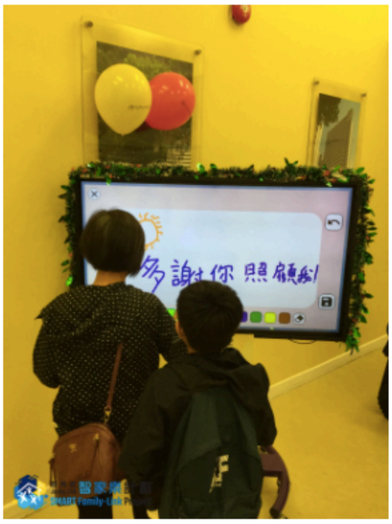

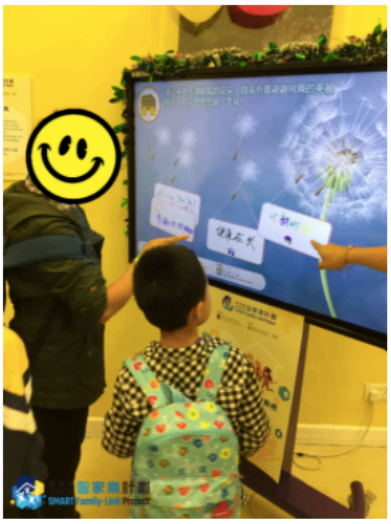

器材	用途
顯示器 [按需要]	顯示器不影響遊戲運作，機構可按實質需要自行配置
Android Tablet	平板電腦上預載遊戲系統，可直接運行遊戲，亦可將畫面輸出至顯示器
Type-C擴充器	轉換至不同制式的輸出

<u>Game E</u>

Promoting positive family communication by gathering happy sayings for family member

Equipment list:

- Screen display
- Computer [with game program pre-loaded]
- Virtual reality device
- Type C extension cable

User experience:

Participants will wear the VR headset and use the hand controls to capture different floating messages of love and appreciation that they see in the space around them for their family members, who will see the messages on the screen display.

Remarks:

VR headsets not recommended for children under 13 years old. For sanitary purposes, disposable VR masks can be purchased and used.

玩法

一位家庭成員戴上虛擬實境眼罩，利用手上的無線控制器在遊戲中收集送給家人的祝福語句。

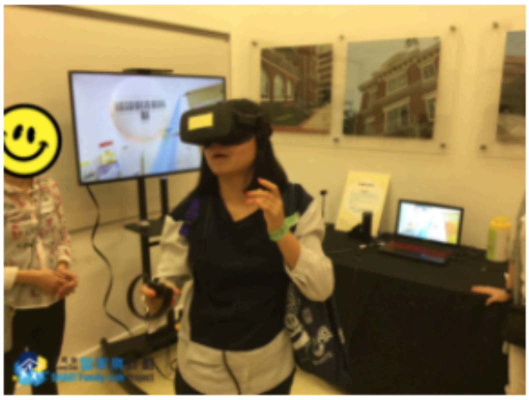

器材	用途
顯示器 [按需要]	顯示器不影響遊戲運作，機構可按實質需要自行配置
電腦 (高階)	電腦上預載遊戲系統，可直接運行遊戲
虛擬實境裝置	遊戲的控制
Type-C擴充器	轉換至不同制式的輸出

Game F

Promoting family communication and happiness by writing or drawing out expressive messages

Equipment list:

- Screen display
- Computer [with game program pre-loaded]
- iPad [with game program pre-loaded]
- Game materials [worksheets with QR codes]

User experience:

Participants write or draw out different messages on various transportation vehicles (such as buses or taxis) pre-printed on worksheets with QR codes. These worksheets are scanned and the vehicle images with different messages are projected onto the screen display and travel around a map.

玩法

一家人用水筆在工作紙上發揮創意，經系統掃描後，工作紙上的交通工作將在大屏幕上行駛。

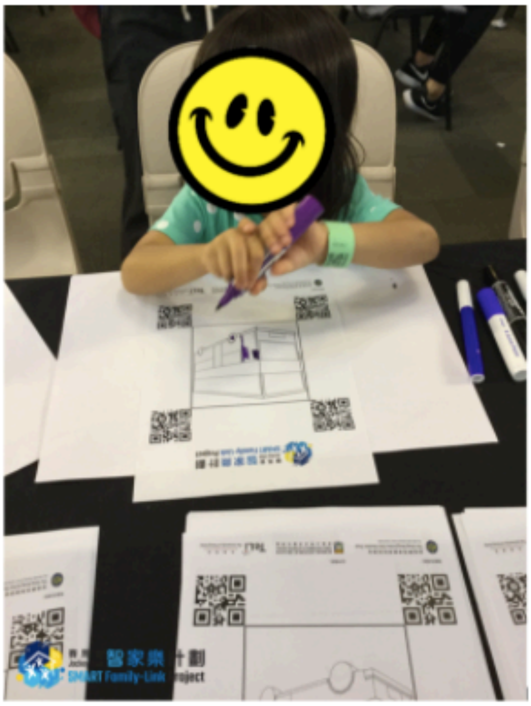

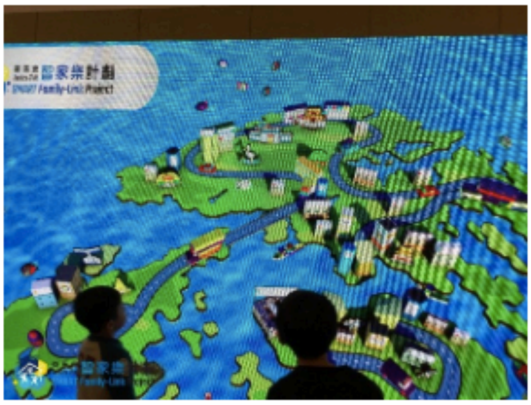

**器材**	**用途**
顯示器 [按需要]	顯示遊戲的畫面，機構可按實質需要自行配置
電腦 [遊戲程式]	建議在機構的電腦上運行遊戲，如有需要，可與智家樂團隊討論
iPad*2 [遊戲程式]	建議在機構的iPad上運行遊戲，如有需要，可與智家樂團隊討論
工作紙 [電子版本]	建議機構按需要預備遊戲用品

<u>Game G</u>

Promoting family communication by taking fun family photos and posing next to a virtual dinosaur

Equipment list:

- Screen display
- Computer [with game program pre-loaded]
- Shooting supplies [includes 4K camera, tripod and green screen]

User experience:

Participants will choose different props, such as swords and capes, and pose in front of a green screen and take a family photo next to a virtual, moving dinosaur. Photos are sent back to them and their respective IFSCs via email.

玩法

一家人站在綠幕前通過電腦合成的方法拍攝一張與恐龍互動的家庭照。

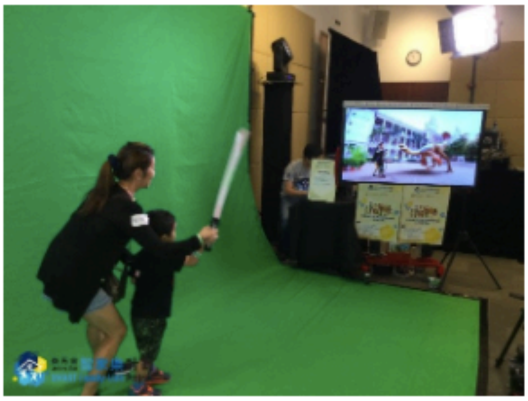

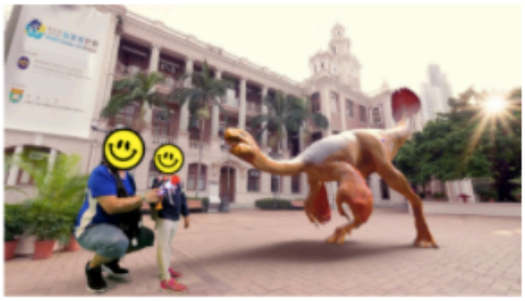

器材	用途
顯示器 [按需要]	顯示器不影響遊戲運作，機構可按實質需要自行配置
電腦 (高階)	電腦上預載遊戲系統，可直接運行遊戲
拍攝用品組裝	包括4K相機、腳架及綠幕組合，用作攝取影象

Game H

Promoting family communication by taking fun family photos with different backgrounds

Equipment list:

- Screen display
- Computer [with game program pre-loaded]
- Shooting supplies [includes 4K camera, tripod and green screen]

User experience:

Participants will choose from different cartoon backgrounds to take a family photo. Photos are sent back to them and their respective IFSCs via email.

玩法

一家人站在綠幕前擺出特別姿勢，配上不同的背景和祝福語句，通過電腦合成的方法拍攝一張別出心裁的家庭照。

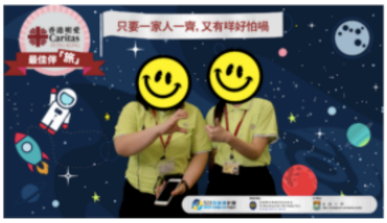

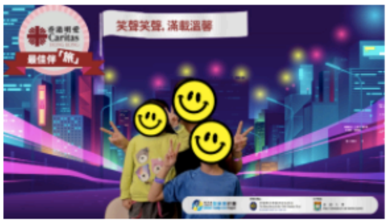

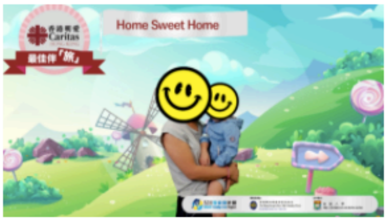

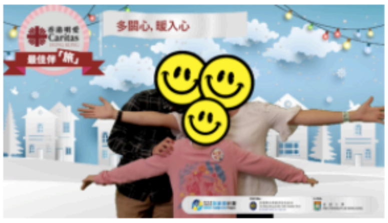

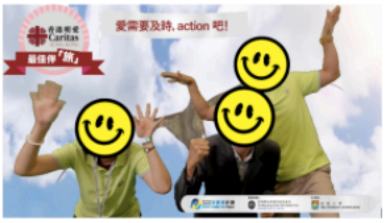

器材	用途
顯示器 [按需要]	顯示器不影響遊戲運作，機構可按實質需要自行配置
電腦 [遊戲程式]	建議在機構的電腦上運行遊戲，如有需要，可與智家樂團隊討論
拍攝用品組裝	包括4K相機、腳架及綠幕組合，用作擷取影象

<u>Game I</u>

Promoting family communication by taking fun family photos framed with different family-related blessings

Equipment list:

- Screen display
- Computer [with game program pre-loaded]
- Shooting supplies [includes 4K camera, tripod and green screen]

User experience:

Participants will use different props and choose a preferred photo frame with different family-related sayings or blessings to take a family photo. Photos are sent back to them and their respective IFSCs via email.

玩法

一家人站在綠幕前配上不同的道具和祝福語句，通過電腦合成的方法拍攝一張別出心裁的家庭照。

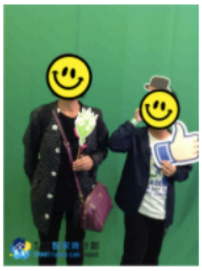

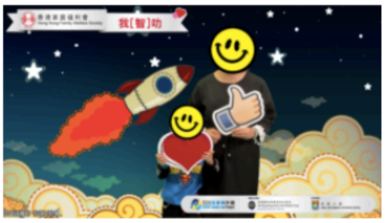

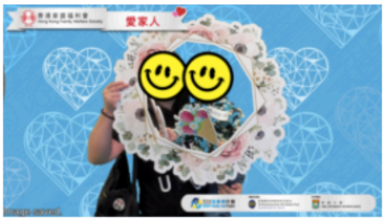

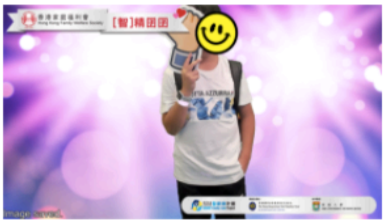

器材	用途
顯示器 [按需要]	顯示器不影響遊戲運作，機構可按實質需要自行配置
電腦 [遊戲程式]	建議在機構的電腦上運行遊戲，如有需要，可與智家樂團隊討論
拍攝用品組裝	包括4K相機、腳架及綠幕組合，用作擷取影象

<u>Game J</u>

Promoting physical activity by competing at timed fitness challenges

Equipment list:

- Screen display
- Computer [with game program pre-loaded]
- Kinect [sensor that captures body movements]

User experience:

One to two family members stand in front of the Kinect sensor and compete with each other to complete the most jumping jacks in 60 seconds.

Remarks:

Game not suitable for frail participants.

玩法

一至兩位家庭成員面向遊戲屏幕，在感應器前同步做出開合大字跳，在限時內挑戰最多成功次數。

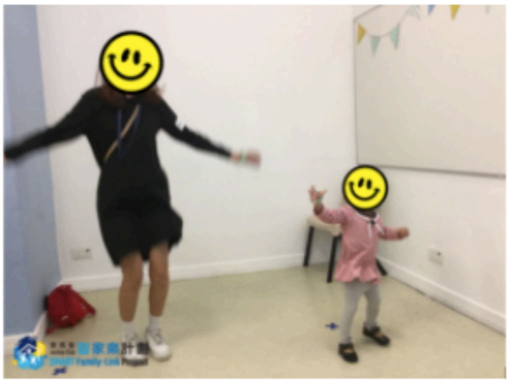

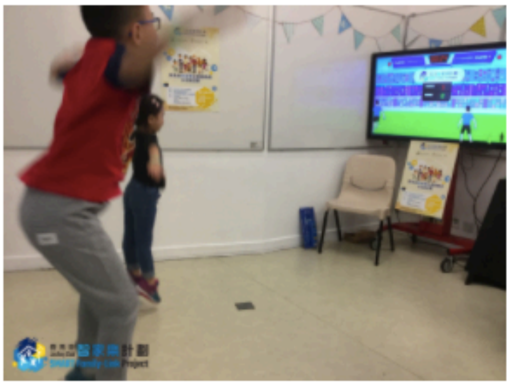

器材	用途
顯示器 [按需要]	顯示器不影響遊戲運作，機構可按實質需要自行配置
電腦 [遊戲程式]	建議在機構的電腦上運行遊戲，如有需要，可與智家樂團隊討論
Kinect	捕捉肢體動作的感應器

Game K

Promoting physical activity and family happiness by playing a basketball shooting challenge

Equipment list:

- Screen display
- Computer [with game program pre-loaded]
- Kinect [sensor that captures body movements]

User experience:

One to two family members stand in front of the Kinect sensor and compete in a basketball shooting challenge by accurately shooting imaginary hoops into the baskets on the screen. The accuracy depends on how straight the arms go up over the head and the speed.

Remarks:

Game not suitable for frail participants.

玩法

一至兩位家庭成員面向遊戲屏幕，在感應器前射籃，在限時內挑戰最高入球分數。

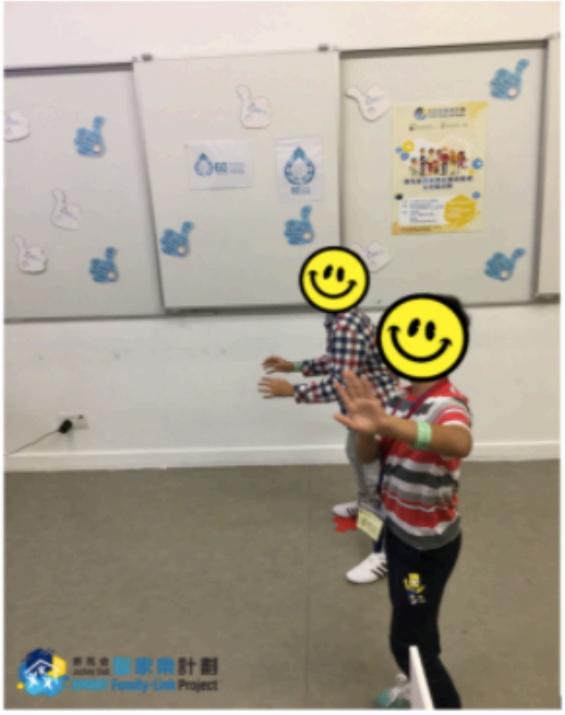

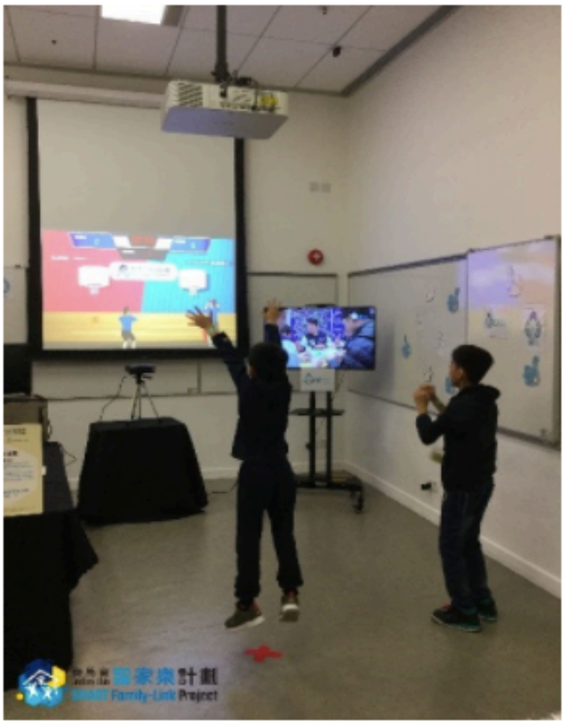

器材

顯示器 [按需要]

電腦 [遊戲程式]

Kinect

用途

顯示器不影響遊戲運作，機構可按實質需要自行配置

建議在機構的電腦上運行遊戲，如有需要，可與智家樂團隊討論

捕捉肢體動作的感應器

Game L

Promoting positive family communication by playing a cooking challenge

Equipment list:

- Screen display
- Computer [with game program pre-loaded]
- Leap [sensor that captures hand movements]

User experience:
One to two family members sit in front of the leap sensor and complete different timed cooking challenges together by mimicking different cooking motions such as chopping and pouring.

玩法

一至兩位家庭成員面向遊戲屏幕，在限時內用雙手的動作按畫面的指示完成菜式。

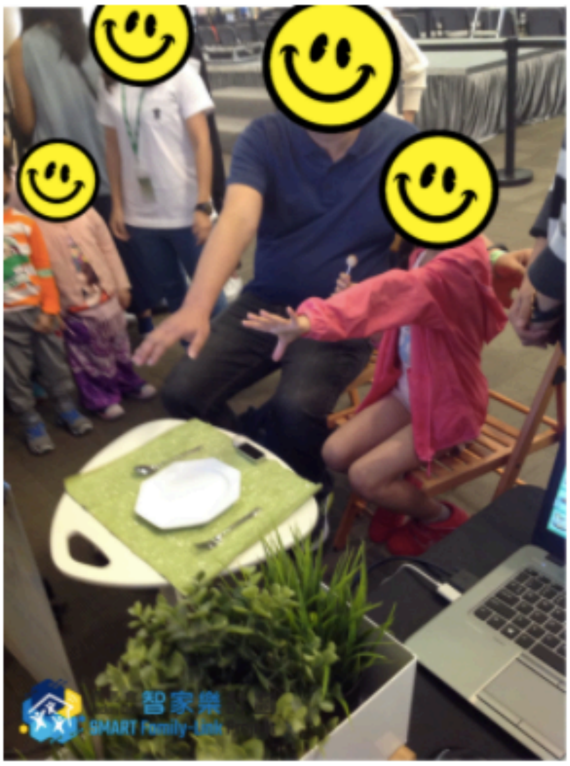

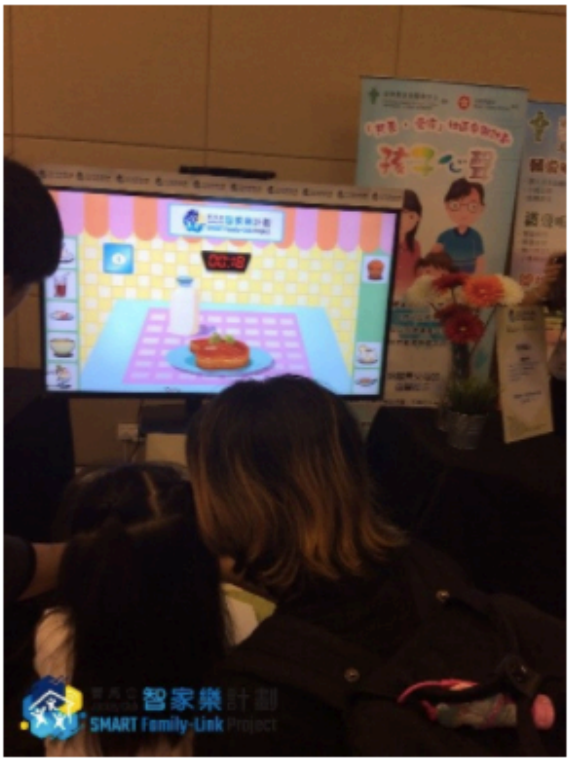

器材	用途
顯示器 [按需要]	顯示器不影響遊戲運作，機構可按實質需要自行配置
電腦 [遊戲程式]	建議在機構的電腦上運行遊戲，如有需要，可與智家樂團隊討論
Leap	捕捉雙手動作的感應器

Game M

Promoting family communication by building a virtual dream home

Equipment list:

- Screen display
- iPad [with game program pre-loaded]
- Game materials [paper cards with QR codes]

User experience:

Participants work together and arrange paper cards with unique QR cards in different configurations to form the ideal, dream home. The iPad scans the QR codes and shows participants the 3D virtual home that they built.

玩法

材料包中的紙片上有特殊條碼，經平板電腦掃描後可變成立體動畫，通過幾位家庭成員討論如何擺放紙片，大家合力共建理想的家。

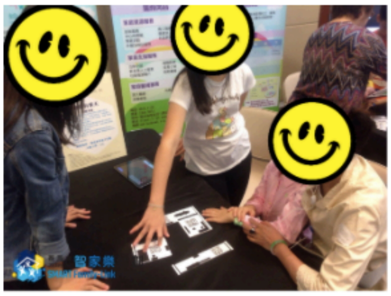

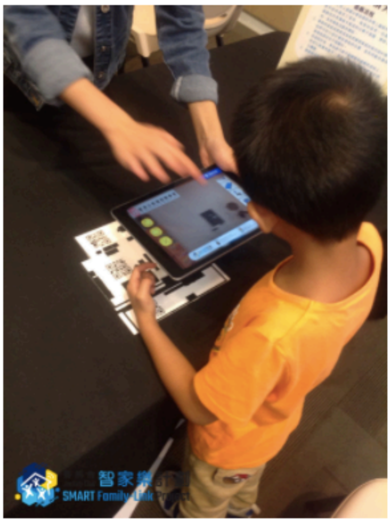

器材	用途
顯示器 [按需要]	顯示器不影響遊戲運作，機構可按實質需要自行配置
IPad [遊戲程式]	建議在機構的IPad上運行遊戲，如有需要，可與智家樂團隊討論
材料包 [電子版本]	建議機構按需要預備遊戲用品

<u>Game N</u>

Promoting family communication and happiness by recording expressive audio messages.

Equipment list:

- Screen display
- Android tablet [with game program pre-loaded]
- Type C extension cable

User experience:

A participant from each family records a message for their family members. These audio messages are automatically transformed into different colored fish, which are added to the aquarium on the screen. Participants can press on their fish to hear the audio message and play it for other family members.

玩法

一位家庭成員對著咪講出想對家人講的說話，系統會將聲音轉化為魚然後加到畫面之中，按魚可以播放錄音。

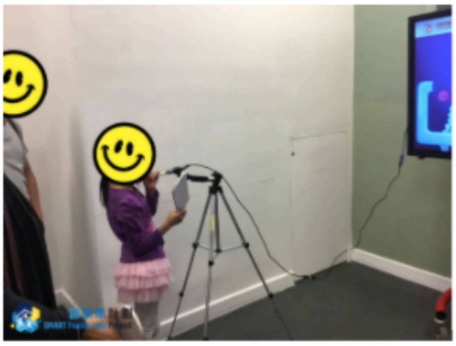

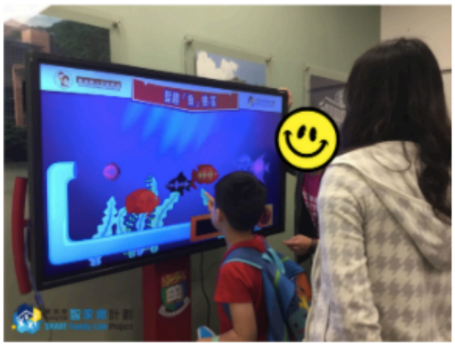

器材	用途
顯示器 [按需要]	顯示器不影響遊戲運作，機構可按實質需要自行配置
Android Tablet	平板電腦上預載遊戲系統，可直接運行遊戲，亦可將畫面輸出至顯示器
Type-C擴充器	轉換至不同制式的輸出
